# The Impact of Canadian Medical Delays and Preventive Measures on Breast Cancer Experience: A Silent Battle Masked by the COVID-19 Pandemic

**DOI:** 10.1177/08445621221097520

**Published:** 2022-04-28

**Authors:** Justine Fortin, Marjolaine Rivest-Beauregard, Clarisse Defer, Mélissandre Leblanc, Lunie Anne Thamar Louis, Carol-Anne Roy, Isabelle Lapierre, Alain Brunet, Marjorie Montreuil, Marie-France Marin

**Affiliations:** 1 Department of Psychology, 14845 Université du Québec à Montréal, Montreal, Quebec, Canada; 2 Centre de recherche de l’Institut en santé mentale de Montréal, 439499 CIUSSS-de-l’Est-de-l’Île-de-Montréal, Montreal, Quebec, Canada; 3 Department of Psychiatry, 5620 McGill University, Montreal, Quebec, Canada; 4 Department of Oncology, Hôpital Maisonneuve-Rosemont 439499 (CIUSSS-de-l’Est-de-l’Île-de-Montréal), Montreal, Quebec, Canada; 5 Department of psychology, 5622 Université de Montréal, Montreal, Quebec, Canada; 6 Department of Psychology, 59310 Université du Québec en Outaouais, Montreal, Quebec, Canada; 7 Patient-partner, Montreal, Quebec, Canada; 8 Department of Nursing, 5620 McGill University, Montreal, Quebec, Canada

**Keywords:** Psychological distress, qualitative research, mental health, oncology, pandemic, recommendations

## Abstract

**Background:**

The COVID-19 pandemic led to the prioritization of breast cancer services towards patients who are currently in treatment or diagnosed with advanced stages of breast cancer, and the self-assessment of both tumor growth and treatment side effects. Alongside the stress associated with cancer itself, delays and complications due to COVID-19 may impact patients’ mental health.

**Purpose:**

To describe the experiences of Canadians living with breast cancer who received a diagnosis and/or treatment during the pandemic, and to identify their recommendations for improving patients well-being during future pandemics.

**Methods:**

Semi-structured interviews were conducted with eighteen women living with breast cancer who also completed the Distress Thermometer questionnaire. The transcripts were analyzed using a descriptive thematic content methodology.

**Results:**

Women who started their breast cancer screening or treatment before the pandemic reported fewer delays and less psychological distress than those who started during the pandemic. Participants reported feeling dehumanized while receiving their medical care, being unable to be accompanied during medical visits, and fearing treatment interruption during the pandemic. Patient recommendations for improving care and psychological support included the presence of family caregivers at consultations to receive the diagnosis and for the first treatment session.

**Conclusion:**

Study findings provide new insights on how healthcare restrictions during the pandemic impacted on patient experiences and their well-being during screening and treatment for breast cancer. The need for cancer nursing practices and care delivery strategies that promote the delivery of compassionate, patient-centred care and the provision of psychological support during future pandemics are identified.

## Background and purpose

As a result of the COVID-19 pandemic, infection prevention and control measures have transformed to protect at-risk populations, such as patients diagnosed with cancer (Burki, 2020; Cortiula et al., 2020). Consequently, many breast cancer clinics in Canada closed their doors during the first year of the COVID-19 pandemic, resulting in a considerable decrease in mammograms and biopsies performed (Farah et al., 2021; Gouvernement du Québec, 2020). These changes led to a reduction of more than 50% in breast cancer related consultations and medical delays across Canada (Gouvernement du Québec, 2020; Yong et al., 2021). In turn, these delays impacted not only the mental health of patients diagnosed with breast cancer (Burki, 2020; Citgez et al., 2020), but also of healthcare workers (Varghese et al., 2021). Oncology nurses were challenged to provide high quality breast cancer care and achieve optimal patient outcomes despite pandemic restrictions on patient access to support and resources (Zeneli et al., 2020). The medical delays led to new nursing challenges that could, in turn, affect patients’ well-being.

Prior to the pandemic, the breast cancer screening phase was already considered stressful, as it involves waiting for a potential diagnosis (Lebel et al., 2003), which may generate concerns about experiencing a life-threatening illness (Smit et al., 2019). However, this stress may be exacerbated by increased wait times due to COVID-19 related restrictions on cancer services (Kutikov et al., 2020). Coupled with pandemic-related stressors (Massicotte et al., 2021), a pre-pandemic study showed that longer wait times before receiving a diagnosis could be increasingly detrimental to the psychological well-being of patients (Howell et al., 2015). During the COVID-19 pandemic, Quebec regulations prioritized cancer services toward patients undergoing treatments, causing further waiting for patients awaiting diagnoses. This worsened existing delays in Canadian breast cancer clinics (Decker et al., 2022; Farah et al., 2021; Gouvernement du Québec, 2020) and could, in turn, further impact patients’ psychological well-being.

A meta-analysis using pre-pandemic data reported that the psychological impact of screening wait times and the receipt of a breast cancer diagnosis can be detrimental to a patient's mental health even before treatments begin (Fortin et al., 2021). During the pandemic, infection control and preventive measures were employed according to the severity of the diagnosis: patients living with stage 4 or recurrent breast cancer were the first to receive treatment, while patients living with stage 0–1 were the last (Farah et al., 2021). To date, the impact of the prioritization of cancer services during the pandemic on Canadian patients living with breast cancer has not been examined. Moreover, emerging COVID-19 regulations led to the modification of treatment plans to prevent patients living with breast cancer from going to the hospital; this included the implementation of online medical follow-ups visits (e.g., medical follow-up visits) (Gill et al., 2020). As a result, these preventive measures were associated with an increase in anxiety and depressive symptoms in patients living with breast cancer (Kim & Kim, 2021). Nonetheless, these regulations were justifiable as these patients are more vulnerable to viruses due to the impact of cancer treatments on the immune system (Burki, 2020; Dai et al., 2020).

Although cancer screenings and treatments have resumed in North America, particular concerns reinforced by the media and/or healthcare teams persist amongst oncology patients, such as fear of appointment cancelation, fear of contracting the COVID-19 at the hospital, and diagnosis and treatment delays (Lum & Tambyah, 2020; Moraliyage et al., 2021; Yong et al., 2021). Medical and nursing teams must address these concerns as they continue to have a psychological impact on patients living with breast cancer. Additionally, oncology professionals should be familiar with patients experiences and implement strategies to ease the psychological burden when needed.

The main objectives of this qualitative study were to describe the experiences of patients living with breast cancer who received a diagnosis and/or treatment during the pandemic, as well as to identify their recommendations to improve patients well-being during current and future time of health crisis. In addition, we measured psychological distress levels for descriptive purposes and to contextualize participant experiences.

## Methods and procedures

This study employed a qualitative descriptive design, allowing us to capture a broad overview of the experience of receiving a breast cancer diagnosis and/or treatment during the pandemic (Doyle et al., 2020). This method recognizes the subjective nature of the domain being studied, for which little is yet known (Doyle et al., 2020).

To enrich the quality of this study, we consulted the expertize of a patient partner who received both her diagnosis and treatments during the pandemic [IL]. She provided input on the conceptualization of the study protocol, and the development of the materials, and the recruitment strategy. Her advice throughout the various phases of the study is discussed in later sections of this paper.

### Participants

The sampling approach used in this study was selected based on the literature. We expected to recruit between 9 and 15 participants (identifying as male, female, or other) who were diagnosed with breast cancer and/or started treatment during the pandemic. This target sample size was inspired by qualitative studies with similar populations, where data saturation was achieved after 9 (Wei et al., 2017), 10 (Landmark & Wahl, 2002) and 15 participants (Ghaemi et al., 2019) were interviewed. However, as we failed to reach data saturation at 15 participants, we recruited a higher number of individuals to provide a better understanding of the participant experience and to meet the study objectives.

Participants were recruited in February 2021 via advertisements through Canadian Community-Based Cancer Support Services, breast cancer Facebook pages, and word of mouth through our patient partner. Participants interested in the study were screened by a research team member [ML, LATL, CAR] by phone to confirm their eligibility.

To be included, the participants needed to 1) speak French, 2) be 18 years of age or older, 3) have been diagnosed with breast cancer and/or treated *during* the pandemic (i.e., when the state of COVID-19 emergency was declared in Canada [March 2020]). The following participants were excluded from participating in the study: participants lacking the technology to attend the interview virtually on a videoconference platform (i.e., if the participant did not have a phone, computer, or tablet), individuals diagnosed or treated for any other type of cancer/health condition, individuals undergoing screening or awaiting a potential breast cancer diagnosis.

### Data collection procedures

Ethics approval was obtained from the Centre intégré universitaire de santé et de services sociaux (CIUSSS) de l’Est-de-l’Île-de-Montréal (CEMTL-24267). After confirming eligibility, participants were asked to sign the information consent form via the secure online platform LimeSurvey. Next, interviews were conducted remotely via the Zoom platform (Archibald et al., 2019; Gray et al., 2020). All interviews (40–120 min per interview) were conducted in February 2021 by the same interviewer to ensure consistency [JF] and were audio and/or video-recorded. The modality of data collection (video vs. audio) was adapted to the participant's preference. After the semi-structured interview, both retrospective and current levels of psychological distress were collected.

Interviews were transcribed by a research team member and then reviewed by two independent research assistants to ensure accuracy and completeness of the ideas presented in the interview. The first author [JF], who also conducted the interviews, revised and finalized all the transcripts.

## Data collection tools

### Interview guide

The interview guide was developed based on different Canadian cancer-related guidelines during the COVID-19 pandemic (see a review of these guidelines: Farah et al., 2021) and input from co-authors with expertize in oncology and qualitative research methods [CD, MM]. Thereafter, revision of the guide by the patient partner resulted in the addition of several questions regarding COVID-related restrictions during medical appointments and psychosocial services. The final interview guide was validated by the patient partner [IL] and all other co-authors. The guide utilized open-ended questions to gather sufficient information to fulfill the main objectives of this study (see Appendix 1).

### Distress thermometer

The validated French version of the Distress Thermometer questionnaire was used to measure subjective distress experienced by the participants as it is often administered to patients diagnosed with cancer in both clinical and research settings. This tool consists of one question and assesses the patient's distress levels in the last seven days on a scale of 0 (no distress) to 10 (extreme distress). First, participants were asked to explain and rate their distress level during their worst breast cancer-related experience, as previously done in the literature (Civilotti et al., 2020; Lally et al., 2020). Then, the participants answered the same question by reflecting on how they felt in the past seven days. A cut-off score of 4 (or over) suggests clinically significant levels of distress (Ownby, 2019). This measure was used for descriptive purposes and to contextualize the interpretation of qualitative results.

#### Qualitative analysis

##### Thematic guide

Analysis was performed using thematic content analysis (Vaismoradi et al., 2015). A thematic guide was developed using both deductive and inductive methods (Bradley et al., 2007). Two research team members [JF, MRB] read the transcripts and coded participant experience according to four major themes: COVID-19 experience, diagnosis-treatment experience, COVID-related delays and preventive measures in healthcare services, and integration of patients’ needs to care and support. Next, the two team members discussed their preliminary analyses with one senior member [MM] to validate the coding method. Following confirmation from the senior member, the two members analyzed three additional transcripts independently in which subthemes emerged inductively. Then, the two members met once more to verify inter-rater reliability across their coding methods (level of agreement 87.5%). If discrepancies between coders occurred, they were resolved through discussion (Chinh et al., 2019). Finally, a thematic guide was created and sent to participants to ensure that their experience was represented within each major theme (Goldblatt et al., 2011). Following approval from the participants and research team, the two research team members completed the remaining analyses deductively. All analyses were conducted in MaxQDA (Kuckartz & Rädiker, 2019). Relevant quotes from the participants were translated from French to English by a member of the research team [JF] and verified by a bilingual member of the research team [MRB] as previously done in the literature (Lajoie et al., 2019).

##### Narrative syntheses

A narrative synthesis was written for each participant to contextualize the data concerning each participant's own experience. The synthesis included narrative descriptions, classification of evidence, commentaries, and interpretations that could build a body of knowledge from the participant's individual experience (Cruzes et al., 2015). Four members of the research team [JF, ML, LATL, CAR] wrote the narrative syntheses according to clear instructions provided by a qualified member of the team [MM]. As previously done in the literature, certain excerpts are presented in the results section to contextualize the themes and subthemes (Montreuil & Carnevale, 2018).

#### Descriptive analyses

Descriptive analyses were conducted to add quantitative input to the qualitative data. All statistical analyses were conducted in SPSS V. 27 to present retrospective and current distress scores obtained with the Distress Thermometer.

## Results

### Sociodemographic characteristics

Eighteen participants identifying as women were included in the final sample with ages ranging from 30 to 64 years old. For further sample characteristics, see Table 1.

**Table 1.** Sociodemographic descriptive (n = 18).

Sociodemographic Descriptive	*N*	*%*
Gender*		
Woman	18	100.0
*Timing of the diagnosis*		
Before pandemic	6	33.3
During pandemic	12	66.7
*Diagnosis Stage*		
Did not know	4	22.2
Stage 1	2	11.1
Stages 1 and 2	2	11.1
Stage 2	3	16.7
Stages 2 and 3	1	5.6
Stage 3	2	11.1
Stage 4	3	16.7
Recurrent diagnosis	1	5.6
*Timing of beginning of treatments*		
Before pandemic	5	27.8
During pandemic	13	72.2
*Medical status at the time of the interview*		
Undergoing treatments	13	72.2
Treatments done	5	27.8
*Marital Status*		
Single	2	11.1
Relationship/Common-law partner	7	38.9
Married	8	44.4
Divorced	1	5.6
*Household situation during the pandemic*		
Alone	1	5.6
With partner	9	50.0
With child(ren)	4	22.2
With partner and child(ren)	2	11.1
With family member(s)	1	5.6
With friend(s)	1	5.6
*Employment situation during the pandemic*		
Working (telework or on site)	2	11.1
Off work due to the pandemic	1	5.6
Off work due to breast cancer	14	77.8
Retired	1	5.6

Notes. *The gender does not refer to the biological sex, but how the participants defined themselves; before the pandemic refers to before March 2020, and during/after the pandemic refers to after March 2020.

The worst moments reported by the participants related to the screening period (*n* = 3), receiving the diagnosis (*n* = 6), fear of the unknown caused by the pandemic regarding their diagnosis/treatments (*n* = 4), diagnosis or treatment changes due to the pandemic (*n* = 3), comorbidity (lung cancer diagnosed during breast cancer treatments) (*n* = 1), and the side effects of the treatments (*n* = 1). According to the Distress Thermometer, women reported high levels of psychological distress at the time of their worst breast cancer-related moment (*M* = 7.06, *SD* = 1.98). At the time of the interview, women reported levels of psychological distress below the clinical cut-off score (*M* = 2.78, *SD* = 2.16). All participants were above the cut-off score at their worst moment, while only six women reported high levels of current distress (scores > 4) at the time of the interview.

### Qualitative results

The thematic content analysis resulted in three major themes, including 1) COVID-related delays and preventive measures for breast cancer diagnosis and treatment, 2) social and medical support, and 3) proposed recommendations. Each of the themes were defined and further divided into subcategories which are discussed below.

### COVID-Related delays and preventive measures in breast cancer diagnosis and treatment

The narrative syntheses identified three trajectories of pandemic-induced delays and preventive measures depending on when participants were diagnosed and started treatment. From this, three groups of participants are presented in Table 2: Group I = Diagnosis and beginning of treatments pre-pandemic while treatments continued during the pandemic, and Group II = Diagnosis pre-pandemic and treatments during the pandemic; Group III = Diagnosis and treatments during the pandemic.

**Table 2.** COVID-Related preventive measures and their impact according to patients’ trajectories (n = 18).

Applied preventive measures	Prioritization of patients who are currently in treatments or diagnosed with advanced stage of breast cancer			Reducing hospital/clinic visits and physical contacts							Canceling non-essential surgeries
Impacts of preventive measures on patients	Delays for screening tests and results	Delays for medical follow-ups	Faster access to treatments	Changes in treatment plan	External follow-ups (by phone)	Self-assessment as medical follow-up	Difficulty obtaining appointments during treatments	Difficulty reaching pivot nurse/resource person	No communication with surgeon before and after surgery	No accompaniment to appointments	Delays for breast reconstruction
Group I (*n* = 5)				X	X	X	X			X	X
Group II (*n* = 2)		X			X	X				X	X
Group III (*n* = 11)	X		X		X			X	X	X	X

**Legend.** Patients’ trajectories: Group I = Diagnosis and beginning of treatments pre-pandemic while treatments continued during the pandemic, Group II = Diagnosis pre-pandemic and treatments during the pandemic, Group III = Diagnosis and treatments during the pandemic.

#### Diagnosis experience

This central theme includes the participants’ experience at the first potential signs of breast cancer (i.e., the time the lump is discovered) until the moment of the official breast cancer diagnosis. This theme also addresses how COVID-19-related delays and preventive measures in healthcare influenced the screening and diagnosis process.

#### Screening phase

According to the participants, no one from Group I and II experienced significant delays between screening and formal diagnosis. The diagnosis was given in person, and only one person was not accompanied during the appointment. The remaining participants were grateful for the moral support during this emotionally difficult appointment.

Women from Group III experienced stressful delays when receiving their screening appointments and diagnoses. One participant was told by her healthcare team that these delays caused her diagnosis to worsen, evolving from stage 0 to stage 1, as she was initially classified as “*low priority*.” The delays led her to report anger and injustice:

“I was not a victim, but I became one because of COVID. A victim of the delays […]. I wasn't revolted to have been diagnosed with cancer; I was revolted to get it during COVID. […] It went from ‘we’ll remove a little bit of your breast’ to ‘we’ll remove both your breasts.’ That was the impact of COVID.”

#### Diagnosis phase

The experience of obtaining a diagnosis during the pandemic was very heterogeneous in Group III depending on the date, specific regulations of the hospitals, or the decisions made by the healthcare team. Four women received their diagnosis in person, of whom only three were allowed to be accompanied. All women who were accompanied acknowledged that they were fortunate to have had the support of their partner during this appointment. On the other hand, a participant who was alone due to COVID-related regulations found her experience very difficult. As she was unable to seek comfort from a partner, she expected “*more empathy*“ from her physician. However, her expectations were not met:

“The distance with the medical team who practically greet you with a five-foot pole [laughs]. I’m exaggerating, but you get that, you know. When I was diagnosed, he [her physician] kind of pushed the Kleenex box towards me. He couldn't do otherwise, he had to keep his distance and all that. That kind of distance is very difficult.”

The rest of the participants from Group III obtained their diagnosis either by phone (*n* = 5), videoconference (*n* = 2), or instant messaging (i.e., Messenger; *n* = 1). Some participants appreciated these modalities as they were grateful to have their support system present during this moment. They also recognized other advantages, such as avoiding exposure to the virus by going to the hospital and reducing the stressful wait time before receiving their results. However, two of the participants who received their diagnosis by phone would have preferred a diagnosis via videoconference to “*humanize*“ the experience. Despite asking, their request was denied. One participant asked if she could use the speakerphone to allow her spouse to hear the information, but the doctor refused due to a fear of being recorded.

#### Reactions to diagnoses

Independently of the pandemic, psychological and behaviural reactions to the diagnosis were diverse across the three groups. When considering the pandemic, four women who were diagnosed with stage 4 breast cancer found it difficult to enjoy “*the last moments of their lives*“ by being confined, isolated, and not being able to “*recharge*“ via physical contact with their loved ones and to engage in meaningful outdoor activities:

“I have an incurable breast cancer, so when you know that you have an expiration date, well, everyone wants to see you, but no one could come see me because we’re in a pandemic.”

#### Treatment experience

The treatment experience theme covers the participant experience from the moment they are waiting for their treatments until they are completed. It also includes how the COVID-19-related delays and preventive measures in healthcare tainted their treatment experience.

#### Surgery and treatments

Regardless of their cancer stage, women from Group I reported that beginning their treatments before the pandemic was an advantage, as they were already in the healthcare system and, therefore, were not impacted by delays. However, the new infection control and preventive measures implemented in March 2020 to reduce the number of hospital appointments led to changes in the treatment plan of some women. Despite these changes, these participants were satisfied with their treatment experience.

As they had no point of reference, it was difficult for some women from Groups II and III to determine whether the delays they experienced were directly related to the pandemic or whether these delays were typical. The treatment experience was positive for one participant who was happy to be on hormone therapy, as it allowed her to stay home during the pandemic, and for two other participants who wanted to receive treatment regardless of the circumstances. However, for many, the preventive measures were harmful to their physical and psychological health. Regarding physical health, a few women (*n* = 5) were required to self-assess their symptoms and side effects after their surgery and during their treatments to determine how well they were recovering. One participant reported that she was not able to determine if her tumor was shrinking and thus, did not notice that it had continued to grow. The latter could have been avoided if follow-up appointments were made via videoconference/in person rather than by phone. Psychologically, the restrictions regarding accompaniment during medical appointments and treatments were the most difficult experience for 12 women. For some, the experience of surgery was stressful and difficult, as they were alone before and after the procedure. In addition, they felt that the process of being dropped off at the hospital was dehumanizing. For others, having someone with them would have been less stressful, as it would have prevented them from being alone when asking questions and receiving information from the healthcare team. During these particular circumstances, several women mentioned taking the initiative to help other patients who appeared distressed without a caregiver in the chemotherapy room. Regarding esthetic procedures, all women (Group I, II, III) who wished to have breast reconstruction were unsure when they would have access to this service due to the low priority nature of the surgery, despite its apparent impact on their well-being and body image.

#### Experience of immunosuppression during the pandemic

The experience of being immunosuppressed during the pandemic was one of the most anxiety-provoking factors among many of the women (*n* = 15) across all groups (I, II, III). Women reported that they did not want to be affected by the COVID-19 virus, not because of its side effects, but because they feared it would prevent them from continuing their breast cancer treatments. For this reason, they used several preventive techniques to reduce anxiety, such as avoiding frequent travel, wearing a mask, and disinfecting their hands. When the healthcare team did not use preventive measures (e.g., going back and forth between COVID-infected areas of the hospital and the breast cancer department, not wearing a mask, discussions about colleagues testing positive for COVID in front of patients), stress amongst patients increased. However, when the healthcare team used preventive measures, participants reported experiencing less stress.

#### Decisions of Canadian leaders regarding patients

Additionally, socio-political issues were raised by some participants (*n* = 7) in each group (I, II, III). Media reports and government decisions were “*contradictory*“ and caused “*anxiety*“ or “*paranoia*“ during all phases of the disease (i.e., breast cancer). Due to this, four participants waited weeks to go to the hospital as they minimized the urgency of consulting a medical professional concerning the lump in their breast. Moreover, many felt that COVID-19 patients were taking over their medical services. In consequence, some questioned whether they were “*really sick*”. The decisions made by the public system related to priority care in times of crisis at the expense of medical care for illnesses such as cancer were also questioned by several participants:

“I was also shocked at the government. There's not just COVID in life! You know, it's not just that. There are people with cancer, there are people undergoing treatments. Hospitals are full every day, and then, all of a sudden, it's just COVID? Wow, that, I was not happy about. So yes, I felt that they [patients with COVID-19 infection] were taking my place.”

### Social and medical support

The social support received by the participants was more “*difficult to obtain*“ due to the social distancing measures during the pandemic. However, many were satisfied with the available alternatives to support them during their hospitalization (e.g., receiving prepared meals, talking to each other by videoconference, talking to each other from the balcony to maintain social distance). All women who did not live alone (*n* = 7) acknowledged that they were “*lucky*“ to always have someone to help them with daily tasks, medical appointments, or to clear their heads. The most widely reported type of support was holding discussions with other patients who were going through similar experiences facilitated by virtual or face-to-face activities offered by Community-Based Cancer Support Services or on breast cancer Facebook groups.

In terms of support from the healthcare team, more than half of the participants felt supported by various professionals. Indeed, many reported that the “*empathy*,” “*availability and quick call returns*,” and “*positive encouragement*“ made them feel “*safe*“ and “*improved their experience*.” The remaining participants did not feel supported by their healthcare team as they would have preferred for the team to provide more answers to their questions (*n* = 6),to be more involved in decisions related to their care (*n* = 4), and to have a pivot nurse who was more available to return calls and do follow-ups (*n* = 4).

### Proposed recommendations

All participants offered recommendations to improve patients’ well-being during COVID-19, which were directed either to the Canadian healthcare system, healthcare teams, or other patients living with breast cancer who were undergoing or soon to undergo the same experience. The most-reported recommendations were related to the importance of recognizing that decision-makers must be more considerate of breast cancer care (both screening and treatment) in times of crisis. Additionally, despite the inevitable preventive measures and delays, it was recommended that healthcare teams remain empathetic. No consistent pattern was found between participant groups (I, II, III) and proposed recommendations. See Table 3 for the proposed recommendations.

**Table 3.** Patients’ strategies and recommendations (n = 18).

Categories	Issue/challenges	Total number of participants who have experienced this issue/challenge (*n*)	Strategies/Recommendations
**Healthcare System Decision and Policy Makers**	Neglecting the importance of social support by not allowing patients to be accompanied during screening, diagnosis or treatment.	15	Allowing to be accompanied during medical appointments or at least at the first important appointment (e.g. the diagnosis, the first treatment appointment).
	Neglecting reconstruction surgeries by classifying them at the same level as cosmetic plastic surgery, thus canceling them during the crisis.	7	Recognizing the impact that breast surgery can have on mental health and body image by not prohibiting it during the crisis.
	Prioritizing patients with COVID during the pandemic, resulting in a sense of injustice for those who also need care.	6	Giving attention to all life-threatening diseases, even in times of crisis.
	Conducting media fear campaigns to show offloading in the health care system.	3	Providing media news that has a positive spin or is more representative of the reality of oncology patients even during times of crisis.
**Community-Based Cancer Support Services**	Younger women did not feel represented in the services offered by the Community-Based Cancer Support Services because they were surrounded by much older women who do not have the same issues and realities.	5	Organizing activities specifically for each age group so that everyone can connect with people who have similar realities to them.
	Patients diagnosed with recurrent breast cancer do not feel represented by the services offered by the Community-Based Cancer Support Services.	4	Have more awareness campaigns and research funds for recurrent breast cancer (stage 4).
**Healthcare teams**	Failure to provide psychosocial support to patients at the time of diagnosis, but also during treatment, when these times can be emotionally difficult.	12	Offering psychosocial services to everyone after diagnosis and during treatment.
	Creating a distance with the patients so that members of the medical team are perceived as less empathic.	9	Taking the time to answer patients’ questions and make sure they are doing well beyond the strictly medical aspects.
	Feeling that the patient is not a stakeholder in their care: no consultation in decision making, no information given and some information omitted.	5	Including the patient in decision making (avoid paternalistic mode).
	The wearing of masks by medical staff creates a dehumanization and a much less proximal experience.	4	Have pictures showing their faces on the medical team members’ uniforms to humanize contact with patients.
	Avoiding discussions on the impact of treatment on fertility and sexuality with women who would like to be informed on the subject and who feel ignored in their needs.	4	Referring all women to oncology sexologists, who can educate them about fertility services and their sexuality during breast cancer if the direct medical team is not comfortable addressing it directly.
	Distancing imposed by the impacts of the pandemic, the atmosphere in the chemotherapy room was somber, not very cheerful. The medical team members did not interact with the patients beyond the medical aspect.	3	Hire comedians/animators to liven up the chemotherapy room and help patients take their minds off of the pre-pandemic atmosphere.
**Other breast cancer patients**	Feeling alone in her experience, despite the presence of family members who do not necessarily understand what the patient is experiencing.	13	Join Facebook groups for breast cancer patients to read testimonies with others who are going through the same thing, but also to get moral support.
	The emotional burden of cancer can leave patients in a dark corner wanting to do nothing.	12	Continuing to do similar activities as before the diagnosis to give meaning to life and not to forget oneself.
	Family and friends, Community-Based Cancer Support Services and medical teams will disseminate information related to breast cancer that may seem negative and impact the well-being of patients.	11	Surrounding yourself with as much positive as possible.
	Disappointment that the medical team is not meeting their individualized needs to improve the diagnostic or treatment experience.	7	Not being afraid to name your needs to members of the medical team and be proactive in the hospitalization experience.

## Discussion

The objective of this study was to describe the experience of patients who received a breast cancer diagnosis or treatment during the pandemic and to inform medical and nursing teams of meaningful ways to improve decision-making and planning regarding the delivery of essential cancer care services. Our results show that the different delays and preventive measures related to the pandemic had both positive and negative impacts on our sample. Mostly, women who were diagnosed and began their treatments before the pandemic (Group I) had less difficult experiences as they were already in the medical system when the pandemic unfolded. However, for participants who were diagnosed (before or during the pandemic) and began their treatments during the pandemic (Groups II and III), the delays and preventive measures caused by the pandemic took the form of psychological and emotional distress, stress, and fear. In our study, psychological distress was mostly related to wait times and uncertainties about whether the pandemic would impact disease progression or potential delays in medical care. Two previous studies reported similar results where higher levels of psychological distress among patients living with breast cancer was associated with fear of disease progression (Chen et al., 2020, 2021).

Moreover, the inability to be accompanied during medical appointments was identified by the majority of our participants. This restriction enhanced fear of unpredictability regarding their diagnosis or first treatment appointments, and highlighted the lack of social support in times of need. This result is supported by the literature showing that body proximity and touch are therapeutic during difficult or uncertain times and can contribute to the reduction of stress and fear (Tabatabaee et al., 2016). In the context of the pandemic, international studies in oncology have also shown that the lack of social support contributes to higher levels of psychological distress in patients living with cancer (see review: Klaassen & Wallis, 2021).

In addition, fear of contracting the COVID-19 virus was reported by all participants. Interestingly, the fear was, for most, related to the fact that their cancer care and treatments would be interrupted if they contracted the virus. These factors were often associated with fear generated by the media and medico-political decisions. A previous study has also shown that the constantly changing government decisions have led to many medical appointments cancelations and the avoidance of screening appointments among patients diagnosed with breast cancer (Kirkegaard et al., 2021). Our study also suggests that such medico-political decisions had a psychological impact on women living with breast cancer during the treatment phase.

It is clear that medical and nursing teams made efforts to adapt their services to the challenges brought by the pandemic. However, unaccompanied mandatory medical appointments were difficult for patients, as they felt that their partner's absence was not always compensated by an empathetic attitude from the healthcare team. However, it is important to note that elevated stress levels experienced by medical teams during clinic visits have been shown to enhance the patients’ perception of feeling dehumanized (Glebocka, 2019). This finding may explain why many of our participants reported being treated like numbers during the pandemic, given the highly stressful pandemic experience for healthcare professionals (da Silva & Neto, 2020).

In addition, self-assessment of treatment side effects and cancer mass evolution was not effective for all participants. Indeed, some women still reported disease progression and avoidable treatment side effects. This medical practice is currently under investigation by researchers, and primary results show that patients can assess themselves as effectively as physicians (Galizia et al., 2018). However, a clear protocol must be given to patients for such measures to be effective (Galizia et al., 2018), which was not the case for the participants in our study. Therefore, it is essential to recognize that the stress experienced by both the healthcare team and service-using patients could significantly influence the effectiveness of assessments (Baumann et al., 2001; Van den Hombergh et al., 2009).

On a positive note, many women reported that they wanted to help other patients by supporting them during difficult medical appointments or by participating in this research study, which allowed them to feel useful. Such empathetic responses correspond with post-traumatic growth, an indicator of resilience that stems from potentially traumatic or highly stressful experiences such as the COVID-19 pandemic and living with breast cancer (Westphal & Bonnano, 2007). Their resilience seems to have guided their will to participate in this study and many of the recommendations directed towards other patients.

## Recommendations for practice

Although the pandemic led to spontaneous medico-political decisions, COVID patients prioritized over cancer patients was at the heart of much discontentment. The most reported issue was the inability to be accompanied during medical appointments. The patients’ caregivers (spouses, friends, parents) were treated as “distance-caregivers,” which prevented patients from receiving emotional, physical, and functional support (Douglas et al., 2016). The importance of social support should not be neglected and should be considered in future implementations from the medical and nursing teams. Additionally, medical and nursing teams should demonstrate their support to patients by adopting a more empathetic approach, which is one major role in nursing practice (Pehrson et al., 2016). Concretely, participants who may have felt dehumanized by their medical team during the pandemic suggested that oncology teams should: wear their uniform with a picture showing their faces; provide patient-centered care by involving the patient in decision-making (e.g., having their partner on difficult appointments); offer psychosocial services to every patient following their diagnosis; and take the time to answer the patients’ questions and to check on the patients’ well-being beyond the medical aspects. Implementing these suggestions in cancer care could be an essential way to respond empathically to patients’ experience.

Recommendations directed towards other patients living with breast cancer revolve heavily around spirituality and optimism, which have been shown to be protective factors for mental health in oncology (Grogan-Henderson, 2003). Although these recommendations were not directed specifically towards healthcare teams, they could nevertheless be incorporated into the resources provided to patients.

Given the similarities that were found across international studies on the experience of screening and treatments for patients living with breast cancer during the pandemic, the recommendations for Canadian medical and nursing practices proposed in this paper could serve as steppingstone for other countries.

## Limitations

This study is not without limitations. The participants are not representative of the breast cancer population as our sample was largely younger than the average age at which women are diagnosed (50 years and older). It is important to note that all participants verbalized that they were highly educated and therefore, were more inclined to find solutions to the various obstacles experienced during their medical experience (e.g., finding psychological services on the Internet). In light of this, it is likely that our results do not accurately capture the experience of women with lower levels of education.

## Conclusion

The results of this study present valuable and important insights into the impact of the delays and preventive measures caused by the pandemic on the well-being of patients living with breast cancer. The experience and recommendations provided by women living with breast cancer should serve as an example for future pandemics or health emergencies, where important decisions need to be made about healthcare services. Additionally, this study highlighted priorities to improve patients’ experiences, support their psychological well-being, and improve cancer outcomes that oncological teams should consider in circumstances beyond the COVID-19 pandemic.

## Appendix 1

### Interview guide

**Table:**

Categories	Questions
**Sociodemographic**	
	Ask the participant to tell a little about him/herself (e.g., age, marital status, employment, impact of COVID on daily life).
**Diagnosis Experience**	
	What was your experience like around your diagnosis?
	What is your specific breast cancer diagnosis
	When were you diagnosed with breast cancer?
	Where were you when you received this diagnosis?
	Were there any delays in your opinion? How was it for you?
	Were you accompanied when you received the diagnosis? If not, why? If yes, was it helpful?
	What psychological services were offered to you at that time? Did you use any of these services, if so, was it helpful?
	Regarding the timing of the diagnosis, were your needs met by the healthcare team? By your loved ones?
**Treatment Experience**	
	When did you begin your treatments, if at all? OR do you know when your treatments will begin?
	In your opinion, are (were) there any delays? How do you feel about these arrangements?
	Can (will) you be accompanied to the clinic? How do you feel about these arrangements?
	What psychological services are available to you to support you during your treatments (have you used any of these services, if so, was it helpful)?
	Regarding treatment, are (were) your needs met by the healthcare team? By your loved ones?
**Personal Needs**	
	What elements (people, services, care) of your experience improved your experience despite the pandemic?
	What elements (people, services, care) of your experience could have been changed to improve your experience despite the pandemic?
**Recommendations**	
	What strategies/recommendations do you think would allow for better psychological support for patients during and after the pandemic?
**Distress Thermometer**	
	From all the experience you shared with me, what was the most difficult/worst moment? Can you explain?
	On a scale of 0 (no distress) to 10 (extreme distress), please state the number that best describes how much distress you have experienced at your worst moment during your breast cancer experience.
	On a scale of 0 (no distress) to 10 (extreme distress), please state the number that best describes how much distress you have experienced in the past week (including today).
**Closing Question**	
	Is there anything else you would like to share with me that could not be covered by the other questions I asked?
